# The time-evolving epileptic brain network: concepts, definitions, accomplishments, perspectives

**DOI:** 10.3389/fnetp.2023.1338864

**Published:** 2024-01-16

**Authors:** Timo Bröhl, Thorsten Rings, Jan Pukropski, Randi von Wrede, Klaus Lehnertz

**Affiliations:** ^1^ Department of Epileptology, University of Bonn Medical Centre, Bonn, Germany; ^2^ Helmholtz Institute for Radiation and Nuclear Physics, University of Bonn, Bonn, Germany; ^3^ Interdisciplinary Center for Complex Systems, University of Bonn, Bonn, Germany

**Keywords:** epilepsy, epileptic network, epileptic focus, seizure, seizure-prediction, seizure-control, electroencephalography, brain dynamics

## Abstract

Epilepsy is now considered a network disease that affects the brain across multiple levels of spatial and temporal scales. The paradigm shift from an epileptic focus—a discrete cortical area from which seizures originate—to a widespread epileptic network—spanning lobes and hemispheres—considerably advanced our understanding of epilepsy and continues to influence both research and clinical treatment of this multi-faceted high-impact neurological disorder. The epileptic network, however, is not static but evolves in time which requires novel approaches for an in-depth characterization. In this review, we discuss conceptual basics of network theory and critically examine state-of-the-art recording techniques and analysis tools used to assess and characterize a time-evolving human epileptic brain network. We give an account on current shortcomings and highlight potential developments towards an improved clinical management of epilepsy.

## 1 Introduction

Epilepsy is the third most common neurological disorder with a worldwide prevalence of about 1% (Beghi et al., 2019; World Health Organization [WHO], 2019). Epilepsy is clinically diagnosed by multiple epileptic seizures or by an epilepsy syndrome or by a single seizure and a probability of at least 60% for further seizures to occur over the next 10 years (Fisher et al., 2005). An epileptic seizure is defined as a transient occurrence of symptoms due to abnormal excessive or synchronous neuronal activity in the brain (Fisher et al., 2005). It can appear due to an acute disease of the brain (e.g., acute symptomatic seizures due to brain abscess), due to systemic disorders (i.e., metabolic disturbances), or as a symptom of a chronic disease, i.e., epilepsy. The disease can be treated sufficiently well in about two-thirds of people with epilepsy (Kwan and Brodie, 2000; Chen et al., 2018), while the other third needs intensive diagnostics and extensive therapy attempts such as non-pharmaceutical interventions (e.g., resective epilepsy surgery, neurostimulation) which in some cases are associated with significant risks or side effects. The people’s willingness to undergo more difficult or risky therapies depends on the burden of the disease being treated. The burden of epilepsy is composed of several factors: comorbidities (Mesraoua et al., 2020), psychosocial impairment, social stigma (Kwon et al., 2022), medico-legal restriction, direct and indirect costs (Strzelczyk et al., 2008; Allers et al., 2015) and—as to be expected—seizures, their treatment and potential complications (Noe, 2019; Mesraoua et al., 2020). The apparent unpredictability of most seizures not only increases the risk of injuries and discomfort, but also imposes loss of autonomy, fear of seizures as well as psychosocial stress (Lang et al., 2022), thereby impacting massively on the quality of life of those affected and their caregivers (Baker et al., 1997; Chiang et al., 2020; Strzelczyk et al., 2023). Thus understanding emergence, propagation, and cessation of epileptic seizures is generally assumed to be crucial to understand the nature of epilepsy, and to understand and establish diagnostic approaches as well as treatment options.

Classification of seizures, epilepsies, and epilepsy syndromes changed over time (Merlis, 1970; Dreifuss et al., 1985; Commission on Classification and Terminology of the International League Against Epilepsy, 1989; Berg and Scheffer, 2011), and an increasingly sophisticated work-up was demanded in clinical daily practice to better understand, characterize, and treat the disease. Moreover, the dichotomy of focal and generalized epilepsy was questioned (Lüders et al., 2009). In the clinical context, the origin of epileptic seizures has been inferred firstly from structural changes and secondly from semiology, i.e., behavioral changes during a seizure. Electrophysiological changes associated with behavioral changes were used as a bridge for diagnosis and referred to as “ictal patterns”. This led to the model of a focal seizure origin and, for a long time, to assignments of involved brain regions to the different zones in presurgical epilepsy diagnosis (symptomatogenic zone, irritative zone, seizure onset zone, epileptogenic lesion, epileptogenic zone, eloquent cortex) (Rosenow and Lüders, 2001). Follow-up data, however, demonstrated that only a portion of subjects treated with focal therapies such as epilepsy surgery (Téllez-Zenteno et al., 2005) or focal brain stimulation achieves long-term seizure freedom (Simpson et al., 2022), which raises doubts about the usefulness of the concept of a focal seizure origin. Furthermore, a similar cerebral lesion does not cause seizures in every subject, so a “proconvulsive” disposition must be suspected.

The epileptic brain should not be viewed as a temporarily disturbed normal brain since it differs from a normal brain in many structural and functional aspects, and seizures take up only a small fraction of a subject’s lifetime. Function and dysfunction of the adaptive dynamical system epileptic brain are interacting processes that cover various time scales and are influenced by various endogenous and exogenous factors. These range from seizures and biological rhythms to treatments with antiseizure medication, neurostimulation, or epilepsy surgery. Moreover, the brain’s dynamics are influenced by its intricate structural complexity; due to its intrinsic plasticity and adaptiveness, dynamics can modify structure (Sporns, 2022). Together, this calls for sophisticated approaches to improve our understanding of the epileptic brain’s complex structure-(dys)functions relationship.

Research over the last decades has demonstrated the excellent suitability of the network approach to the complex system brain in health and disease (Bullmore and Sporns, 2009; 2012; Avena-Koenigsberger et al., 2018). The explicit time-dependence of the epileptic brain, however, required an additional change in perspective from a static to a time-evolving network. We here review this novel perspective, its concepts, definitions, and accomplishments, and discuss possible translations into clinical practice.

## 2 Conceptual considerations

In her seminal work, Susan Spencer considered *a network to be a functionally and anatomically connected, bilaterally represented, set of cortical and subcortical brain structures and regions in which activity in any one part affects activity in all the others. The essential operational component of this definition is the observation that vulnerability to seizure activity in any one part of the network is influenced by activity everywhere else in the network, and that the network as a whole is responsible for the clinical and electrographic phenomena that we associate with human seizures. Implicit in this idea is that the seizures may entrain this large neural network from any given part, such that it becomes irrelevant to discuss the “onset” of seizures in any specific part of the network. In other words, the electrical hyperexcitability associated with seizure activity reverberates within the neural structures of the network, which operate together and inextricably to culminate in the eventual expression of seizures* (Spencer, 2002).

BOX 1 **The number of papers on epileptic brain network published during the past three decades (sourced via Google Scholar using the keywords “epileptic network” and “epileptogenic network”).**

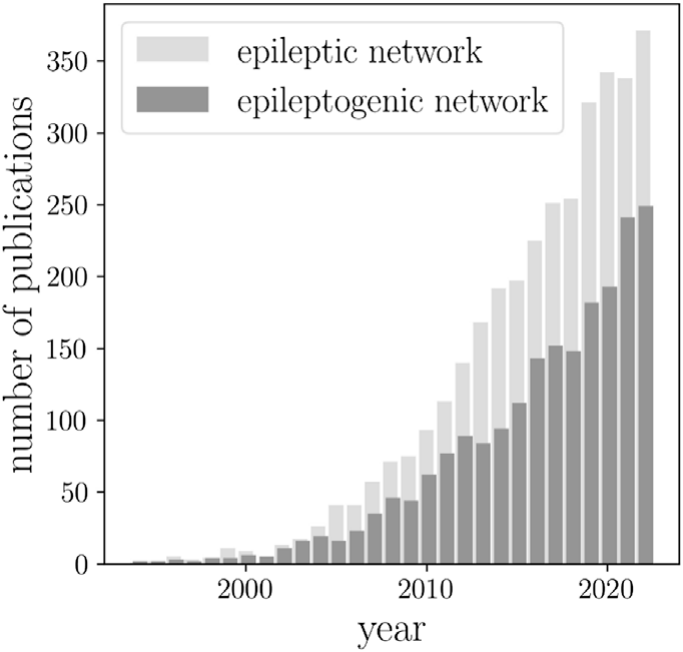

Since then, epileptic brain network studies (sometimes also referred to as an epileptogenic network) increased by almost a factor of 30 (Box 1). In 2010, the term *network* was included in the revised terminology and concepts for organization of seizures and epilepsies of the International League against Epilepsy (Berg et al., 2010). This highlighted the transition from the old concepts of an epileptic focus and various cortical zones involved in epilepsy (Rosenow and Lüders, 2001) to an epileptic network spanning lobes and hemispheres. Today, epilepsy is considered a network disease. The progress made in this highly interdisciplinary research field has been repeatedly summarized in a number of reviews [see, e.g. (Richardson, 2010; Kramer and Cash, 2012; Laufs, 2012; Engel et al., 2013; van Diessen et al., 2013; Chiang and Haneef, 2014; Stam, 2014; van Mierlo et al., 2014; Bernhardt et al., 2015; Bartolomei et al., 2017; Zijlmans et al., 2019; Gil et al., 2020; Royer et al., 2022a)]. A large number of studies provided increasing evidence for an epileptic brain network to differ from healthy ones, both structurally (Whelan et al., 2018; Hatton et al., 2020; Larivière et al., 2020; Sisodiya et al., 2022) and functionally (Chavez et al., 2010; Horstmann et al., 2010; Zhang et al., 2011; Richardson, 2012; Caciagli et al., 2014; Chiang et al., 2015; Foit et al., 2020; Pegg et al., 2020; Slinger et al., 2022). Moreover, studies demonstrated that the network approach allows for an improved understanding of the dynamics of seizures [both generalized and focal (Schindler et al., 2007b; Ponten et al., 2007; Kramer et al., 2008; Schindler et al., 2008; Ponten et al., 2009; Zaveri et al., 2009; Kramer et al., 2010; Kuhnert et al., 2010; Wilke et al., 2011; Bialonski and Lehnertz, 2013; Varotto et al., 2012; Burns et al., 2014; Geier et al., 2015a; Geier et al., 2015b; Zubler et al., 2015; Lopes et al., 2018; Rungratsameetaweemana et al., 2022)] and of the complex interplay between the epileptic process and physiologic activities (Kuhnert et al., 2013; Yaffe et al., 2015; Garcia-Ramos et al., 2016; Vecchio et al., 2016; Steiger et al., 2017; Tailby et al., 2018; Yang et al., 2018; Zaveri et al., 2020; Mutti et al., 2022; Roliz and Kothare, 2022), possibly mediated by the same neural substrate.

Some studies [see, e.g., Zijlmans et al. (2019) for an overview] attempted to integrate the concepts of an epileptic focus and of the aforementioned cortical zones into the concept of an epileptic network by considering the former ones as hubs or hub-like structures, i.e., strongly connected (functionally and/or structurally) network components that significantly impact on the network. The characteristic of being strongly connected, however, is only one of many other properties (cf. Section 3.2) that emphasize a network component as important for both structure and function of an epileptic brain network. Studies going beyond hubs or hub-like structures attribute a rather subordinate role to the epileptic focus and zones for seizure dynamics and for the many (patho-)physiologic phenomena seen in between seizures (Geier et al., 2015a; Geier et al., 2015b; Geier and Lehnertz, 2017b; Geier and Lehnertz, 2017a; Bröhl and Lehnertz, 2019; Rings et al., 2019b; Fruengel et al., 2020; Bröhl and Lehnertz, 2022).

In 2003, John Milton and Peter Jung considered epilepsy as a *dynamic disease* and defined an epileptic system as *a dynamic, ever-changing and evolving, distributed system of neurons that controls the onset, propagation, and arrest of epileptic seizures* and coined the term *evolving epileptic network* (Milton and Jung, 2003). This concept initially received only little attention. It regained interest, however, with the fusion of time-series-analysis techniques and graph-theoretical concepts necessary to investigate evolving (or time-dependent/temporal/multiplex/multilayer) networks (Holme and Saramäki, 2012; Boccaletti et al., 2014; Kivelä et al., 2014; Lehnertz et al., 2014; Muldoon and Bassett, 2016) and with the development of techniques that allow recording and storage of spatially extended brain dynamics assessed over extended periods of time (days to weeks and beyond).

## 3 Techniques to assess and characterize a time-evolving brain network

In a brain network and depending on the chosen spatial scale, a vertex may represent a single cell (e.g., neuron), a group of cells (e.g., cortical columns), or a brain region (e.g., parcellated area) and an edge some connection between vertices. If an edge represents a physical/anatomical connection (single synapses, fiber bundles, or groups of fiber bundles), then the network is referred to as *structural network* (Park and Friston, 2013). If an edge represents some functional interaction between vertices (characterized by the interaction’s strength, direction, and functional form), then the network is called a *functional network* (Park and Friston, 2013).

### 3.1 Recording the brain’s structure and dynamics

Various imaging and recording techniques can be used to assess structure and dynamics of a time-evolving epileptic brain network on different spatial and temporal scales and with different levels of invasiveness (see Table 1).

**TABLE 1:** Structural and (indirect/direct) functional imaging and recording techniques predominantly used to assess structure and dynamics of a time-evolving epileptic brain network (principle: physical mechanisms underlying the measurement; information: information provided by images/time series; n.a. not applicable).

Structural imaging techniques			
	principle	information	resolution
Computed tomography (CT)	measure attenuation of X-rays by different tissues; uses ionizing radiation	mean attenuation (relative to water) of tissues in a given pixel/voxel (greyscale-coded)	spatial: $∼0.5{\text{mm}}^{3}$ temporal: n.a.
Magnetic Resonance Imaging (MRI)	measure magnetization properties of atomic nuclei (mostly hydrogen) employing the techniques of nuclear magnetic resonance; uses strong magnetic field, gradient fields, and radio waves	magnetization properties (e.g., proton density, different relaxation times, diffusion characteristics) of tissues in a given pixel/voxel (greyscale-coded)	spatial: $∼1{\text{mm}}^{3}$ temporal: n.a.
indirect functional imaging techniques			
Positron Emission Tomography (PET)	measure changes in metabolic processes and in other physiological activities with radioactive substances (positron emitter)	changes of processes/activities (e.g., cerebral blood flow) per time unit in a given pixel/voxel (color-coded statistical maps)	spatial: 5–10 mm (pixel size) temporal: 5–10 s
Functional Magnetic Resonance Imaging (fMRI)	measure brain activity by detecting changes in magnetization properties (T2* relaxation time) of hydrogen associated with blood flow (blood oxygenation-level dependent (BOLD) effect); assumes coupling between cerebral blood flow and neuronal activation	BOLD time series; difference between time series recorded during activation and control condition across the brain or from a specific brain region (color-coded statistical maps)	spatial: 3–4 mm (pixel size) temporal: s
Functional Near-Infrared Spectroscopy (fNIRS)/Diffuse Optical Tomography (DOT)	measure brain activity by detecting attenuation (absorption) of near-infrared light associated with blood flow (BOLD effect); assumes coupling between cerebral blood flow and neuronal activation	time series of relative concentration changes in O_2_HB and HHb; difference between time series recorded during activation and control condition across the brain or from a specific brain region (color-coded statistical maps)	spatial: 10–20 mm (pixel size) temporal: s
direct functional imaging techniques			
Electroencephalography (EEG)	measure the spontaneous electrical activity (net effect of ionic currents) of the brain as voltage fluctuation at multiple sensors (electrodes) placed on the scalp (non-invasive EEG) or intracranially (invasive EEG)	multiple time series of voltage fluctuations	spatial: sensor space: distance between sensors
			source space: few mm
			temporal: ms
Magnetoencephalography (MEG)	measure the spontaneous magnetic activity of the brain produced by electrical currents occurring naturally in the brain using very sensitive sensors (e.g., superconducting quantum interferences devices) placed over the head	multiple time series of field fluctuations	spatial: sensor space: distance between sensors
			source space: few mm
			temporal: ms

Among the structural neuroimaging techniques, x-ray computed tomography (CT) (Ginat and Gupta, 2014; Pelc, 2014) and magnetic resonance imaging (MRI) (Mori et al., 2005; Atlas, 2009) allow the non-invasive study of the whole brain at different levels of spatial resolution, ranging from millimeter-sized voxels to cortical areas and beyond. During the recording, time-dependent structural changes (Fjell and Walhovd, 2010) are assumed to be negligible. Due to a comparatively better differentiability of gray and white matter, particularly MRI and diffusion MRI (Bammer, 2003) are often used to probe the topological organization of the brain (Duncan, 2009; Engel et al., 2013; Duncan et al., 2016; Whelan et al., 2018; Sotiropoulos and Zalesky, 2019; Yeh et al., 2021; Zhang et al., 2022). In a structural network (also referred to as structural connectivity (Horwitz, 2003) or structural connectome), discrete regions of gray matter represent a network’s vertices and white matter fibers a network’s edges. In order to identify such network constituents, a large number of approaches is employed to parcellate the brain (Eickhoff et al., 2018; Amunts et al., 2020; Bijsterbosch et al., 2020; Royer et al., 2022b) and to identify and characterize white matter fibers (Mori and Van Zijl, 2002; Jeurissen et al., 2019). The variety of techniques requires appropriate approaches to verify and increase the reproducibility of results (Bonilha et al., 2015; Welton et al., 2015; Zalesky et al., 2016; Roine et al., 2019; Lawrence et al., 2021; Alemán-Gómez et al., 2022; Borrelli et al., 2022; Seider et al., 2022; Charvet, 2023; Madole et al., 2023).

Among the functional neuroimaging techniques, positron emission tomography (PET) (Phelps and Mazziotta, 1985; Juhász and Chugani, 2003; Muehllehner and Karp, 2006; Vaquero and Kinahan, 2015; Watabe and Hatazawa, 2019; Seshadri et al., 2021), functional magnetic resonance imaging (fMRI) (Detre, 2004; Buxton, 2013), functional near-infrared spectroscopy (fNIRS) (Jöbsis, 1977; Villringer and Chance, 1997; Ferrari and Quaresima, 2012; Nguyen et al., 2018; Chen et al., 2020), or (high-density) diffuse optical tomography (DOT) (Eggebrecht et al., 2014; Wheelock et al., 2019) can provide non-invasive indirect access to transient neural activity (time scale: some 10 to some 100 milliseconds) by measuring transient changes in cerebral blood flow and/or metabolic processes (time scale: up to some 10 s) assumed to be related to neuronal activation [neurovascular coupling hypothesis; Roy and Sherrington (1890); Huneau et al. (2015); Kaplan et al. (2020); Drew (2022)]. While providing whole-brain coverage, the temporal resolution of these techniques ranges in the order of seconds and is, in general, dictated by the respective imaging device. An exception is MR-encephalography (Hennig et al., 2021), which also allows whole-brain coverage in 100 ms and with a spatial resolution that compares to the other techniques (few millimeters) (Rapisarda et al., 2010; Torricelli et al., 2014; Chaimowet al., 2018). Statistical dependencies (correlation, cross-correlation) between recorded signals from pairs of vertices (from voxels to cortical areas) are often used to define edges of a functional network [also referred to as functional connectivity (Friston, 2011) or functional connectome].

Direct non-invasive access to both transient and ongoing neural activities is achieved with electroencephalography [EEG (Niedermeyer and Lopes da Silva, 2005)] and with magnetoencephalography [MEG (Baillet, 2017)], both of which allow whole-head coverage and have high temporal resolution (few milliseconds). So far, only EEG allows for the continuous recording of brain dynamics over extended periods of time [days to weeks and beyond (Lehnertz et al., 2017; 2021; Milne-Ives et al., 2023)]. This may also be achieved in the future with further improvements of optically-pumped magnetometer MEG systems (Hill et al., 2019; Boto et al., 2021; Pedersen et al., 2022; Hillebrand et al., 2023). An additional access to the brain’s dynamics at the meso- ( $\approx 10^{5}$ neurons) and micro-scale (single neurons) can be achieved with invasive (intracranial) EEG (Parvizi and Kastner, 2018; Mercier et al., 2022; Soloukey et al., 2023). Although this approach provides the highest signal-to-noise ratio, it has limited spatial coverage of the brain and is limited to some brain pathologies (such as epilepsy) as it requires electrodes to be implanted (temporarily or chronically) onto the surface (electrocorticography) and/or within the brain (stereo-EEG, local field potentials, single-neuron activity). For EEG recordings, the choice of the reference electrode is a notoriously ill-defined problem (Hagemann et al., 2001; Yao et al., 2005; Zaveri et al., 2006; Rummel et al., 2007; Qin et al., 2010; Geier and Lehnertz, 2017b; Anastasiadou et al., 2019; Yao et al., 2019; Babiloni et al., 2020; Delorme, 2023).

EEG/MEG-based techniques allow to capture a wide spectrum of physiological and pathophysiological activities on various time scales and as such, often require other, more sophisticated time-series-analysis techniques (see Section 3.2) to characterize interactions between the sampled brain regions. Properties of interactions are then used to define edges of a functional network whose vertices are usually associated with sensors (EEG-electrodes, MEG-magnetometers) that capture the dynamics of the sampled neuronal substrate. The number of network vertices may range from a few ten to a few hundred, depending on recording technique and research question. Instead of estimating properties of interactions in *sensor-space*, it has been proposed to do so in *source-space* to overcome the problems of volume conduction (EEG) and field spread (MEG) [see, e.g., van Mierlo et al. (2019); Koutlis et al. (2021); Sadaghiani et al. (2022); Chiarion et al. (2023) and references therein]. Nevertheless, localizing the sources of EEG/MEG activities in the brain constitutes an inverse problem that lacks a unique solution (von Helmholtz, 1853) and source-space-based network approaches continue to be critically discussed (Colclough et al., 2016; Palva et al., 2018; Koutlis et al., 2021; Adamovich et al., 2022; Pourmotabbed et al., 2022; Schaworonkow and Nikulin, 2022; Hatlestad-Hall et al., 2023).

As with the structural neuroimaging techniques, the variety of approaches and methods used in functional network research requires appropriate approaches to verify and increase the reproducibility of results (Niu et al., 2013; Zuo et al., 2014; Zuo and Xing, 2014; Geng et al., 2017; Adamovich et al., 2022; Bottino et al., 2022; Kato et al., 2022; Rolle et al., 2022; Wang et al., 2022; Helwegen et al., 2023). This applies even more to the fusion of structural and functional imaging techniques (Luat and Chugani, 2008; Aiello et al., 2016; Babaeeghazvini et al., 2021; Wu et al., 2021) as well as to the combined use of different functional imaging techniques (e.g., EEG-fNIRS or EEG-fMRI) that is often pursued to balance the disadvantages of one method with the advantages of another method (Machado et al., 2011; Nguyen et al., 2012; Centeno and Carmichael, 2014; Obrig, 2014; Pittau and Vulliemoz, 2015; Tousseyn et al., 2015; Abreu et al., 2018; Sanz-Garcia et al., 2018; Rizkallah et al., 2020; Anderson et al., 2021; Bernabei et al., 2021; Uchitel et al., 2021; Ikemoto et al., 2022; Li et al., 2022; Mulert and Lemieux, 2023).

### 3.2 From observations to a functional brain network

**FIGURE 1:**
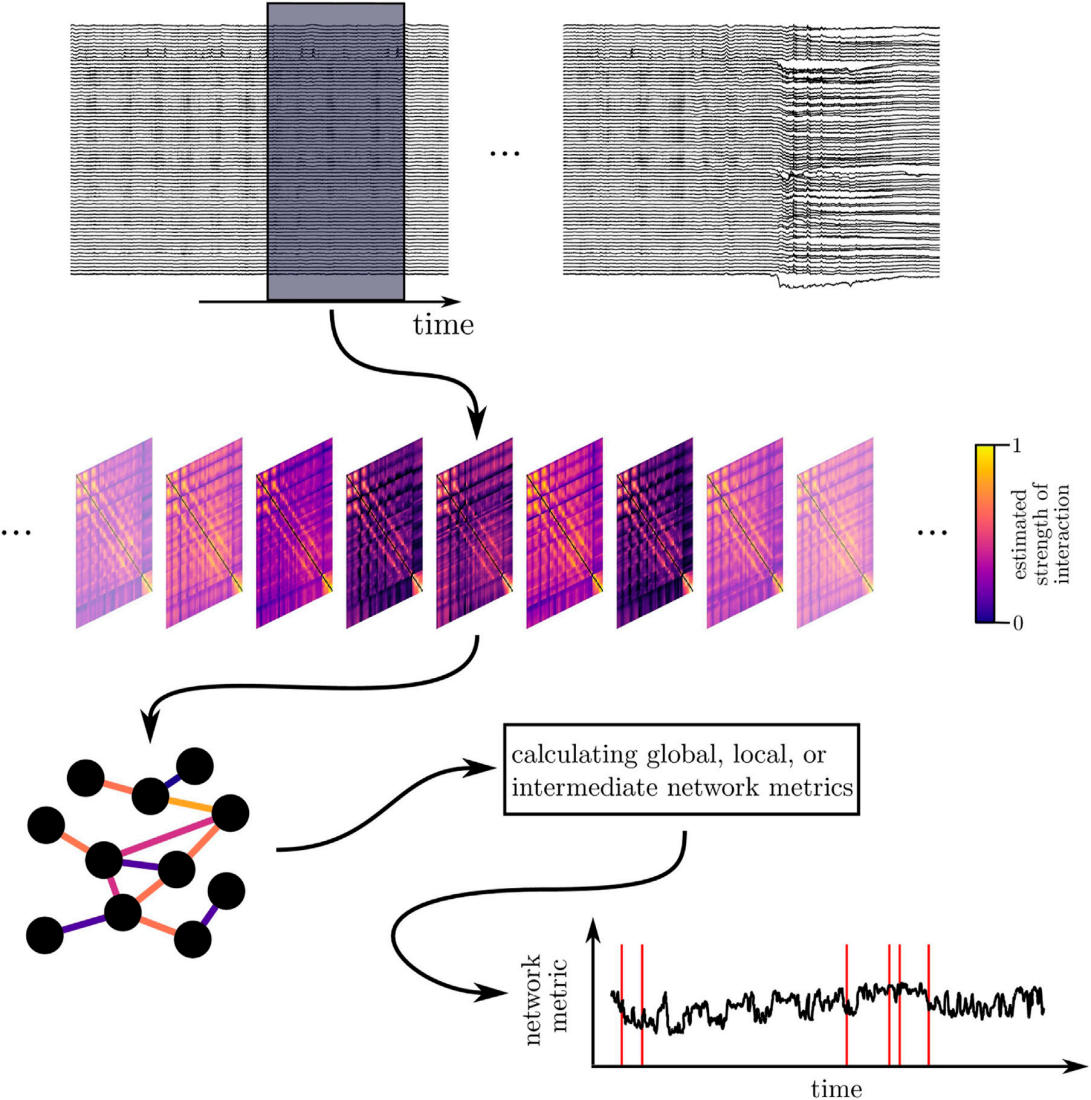
Schematic of deriving and characterizing a time-evolving epileptic brain network. Top: sliding-window analysis: long-lasting multichannel EEG recordings are segmented into successive (non-)overlapping windows. Middle: time-dependent sequence of interaction matrices (functional brain networks): each matrix contains estimates of an interaction property (here: strength of interaction) calculated from EEG data within a given window for all pairs of sampled brain regions. Bottom: a network analysis approach renders a time-dependent sequence of a network metric, which is then subject to further analyses. Red lines exemplarily indicate occurrence of epileptic seizures.

Having recorded the dynamics of various brain regions as multivariate time series, a common way to construct a functional brain network consists of associating network vertices with sampled brain regions and network edges with properties of an interaction (strength, direction, functional form) between pairs of brain regions derived from their dynamics (cf. Figure 1).

The latter ansatz is often pursued in cases where a perturbation-based approach (*actio est reactio*) is either unfeasible or not constructive. Properties of an interaction can then be estimated with diverse linear and nonlinear, bi- and multivariate time-series-analysis techniques grounded in statistics (Rodgers and Nicewander, 1988; Hamilton, 2020), nonlinear dynamics (Kantz and Schreiber, 2003; Datseris and Parlitz, 2022), synchronization theory (Arnhold et al., 1999; Pikovsky et al., 2001; Stankovski et al., 2012; Rosenblum and Pikovsky, 2023), statistical physics (Tabar, 2019), and information theory (Hlaváčková-Schindler et al., 2007), among others.

Given that interactions can manifest themselves in many (also conceptually) different ways (such as the diverse forms of synchronization, flow of information, or similarity) and since there is no *one-fits-all* analysis technique for all types of data or interactions (Pereda et al., 2005; Kreuz et al., 2007; Wendling et al., 2009; Höller et al., 2017), the choice of a time-series-analysis technique is often dictated by the specific research question. Examples of some of the available techniques to estimate properties of interactions are listed in Table 2. Also, depending on the employed recording technique (alongside with the sampling interval; see above), time series can cover various time scales of brain dynamics and include signal properties reflecting different physiological and pathophysiological phenomena. Especially for EEG and MEG recordings, the temporal resolution allows for the separation of the signal into various frequency bands of neural oscillations.

**TABLE 2:** Examples of time series analysis techniques used to characterize properties of an interaction based on different signal characteristics. The strength of an interaction quantifies the level of interdependence between two brain regions. Estimators for the strength of an interaction are predominantly based on the idea that more (abstractly) similar dynamics reflect a stronger coupling between regions. The direction of an interaction assesses which of the two interacting brain regions is driving the other. Estimators for the direction of an interaction are usually based on assumptions about cause and effect in the larger system respectively on models for the temporal evolution of the regions’ dynamics. The functional form of interaction describes the relationship between two brain regions as a mathematical model. Estimators for the functional form of an interaction have the dual task of setting up an appropriate model for the involved interdependencies and of appraising model parameters, which typically requires strong assumptions and in-depth knowledge of the involved dynamics.

Property of interaction	Signal characteristic	Analysis technique
strength	amplitude	(cross-)correlation
	phase	mean phase coherence
	information content	mutual information
	state space	nonlinear interdependence
direction	amplitude	Granger causality
	phase	evolution map approach
	information content	transfer entropy
	state space	nonlinear interdependence
functional form	phase	phase dynamics reconstruction

However, spectral limits of brain activity often associated with these frequency bands might vary in time or between brain regions. It also might not be useful to investigate frequency bands without discernible power, while a broader perspective may include otherwise unnoticed phenomena (Osterhage et al., 2007a; Frei et al., 2010; Gerster et al., 2022). In addition, the human brain has to be regarded as an open, dissipative, and adaptive dynamical system and is inherently non-stationary. Most methods to characterize properties of interactions, however, require the system to be (at least approximately) stationary to yield robust and reliable characterizations. Thus, time series of recordings of brain dynamics are typically cut into segments of appropriate duration whose choice is usually a compromise between the required statistical accuracy for the characterization and approximate stationarity within a segment’s duration [see Lehnertz et al. (2017) for details]. Together, experimental conditions and handling of the brain’s non-stationarity result in the investigation of either carefully-selected segments (possibly influencing findings with selection bias) or of sequences of (non-) overlapping segments or windows (moving-window approach).

Estimates of properties of an interaction can be affected by a number of influencing factors that may arise from specifics of the applied recording techniques, specifics and uncertainties of the various time-series-analysis techniques (cf. Box 2) or due to unavoidable noise contamination. To at least minimize these influences and to improve reliability of estimates, the surrogate approach from statistical hypothesis testing can be employed. This bootstrapping approach begins with formulating an appropriate null hypothesis (Efron, 2004), which specifies properties of influencing factors that might lead to the results of an analysis (cf. Box 3).

BOX 2 **The majority of estimators for the strength of an interaction increase non-proportional (i.e., non linear) with an increase of the coupling strength (assuming one knows the true mechanisms for an interaction between two systems (brain regions)). On the one hand, this depends very much on the systems under investigation, but also the choice of the time-series-analysis technique plays an important role. Even in the case of strong coupling, amplitude-based (blue) or information-theory-based estimators (purple) may indicate a low or medium strength of interaction. In contrast, a phase-based estimator (red) already reaches its maximum value. In the first case, the two systems would be interpreted as weakly interacting or even independent, while the second case would indicate a stronger interaction or even a complete alignment (synchronization).**

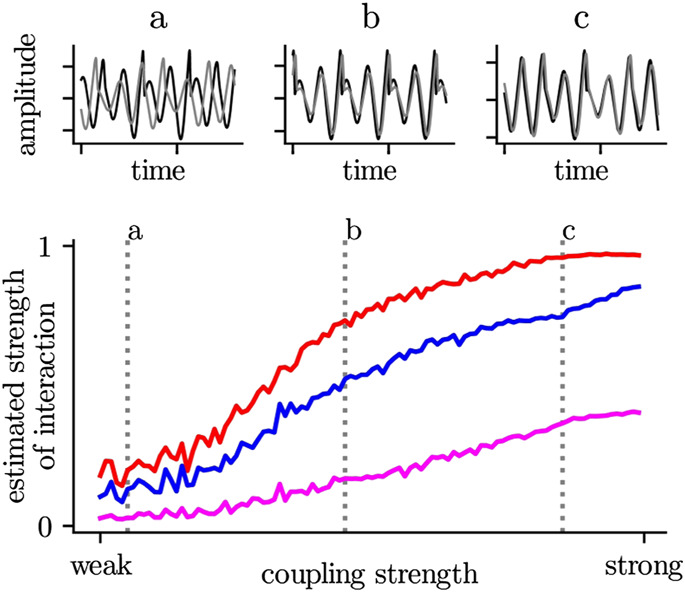

BOX 3 **A null model is a statistical model that is built on a null hypothesis. It makes an assumption about a fact, which can be evaluated by way of a statistical test. Using a null model one can test whether analysis results are purely coincidental and cannot be traced back to specific influencing factors. If the test confirms the null model, this is not a proof for its correctness. It only leads to a rejection of the hypothesis of the previously assumed dependence on the influencing factors. A null model can not be proven, but only disproved. With suitable methods, so-called surrogates can be created from the original time series (top) or from networks (bottom), whereby the influencing factor to be investigated is hidden and factors to be investigated are masked out. If analysis results for the original data differ from those for a sufficiently large number of surrogates, then the null hypothesis can be rejected with ascertainable certainty. The investigated influencing factor then plays a significant role and must be taken into account when interpreting findings.**

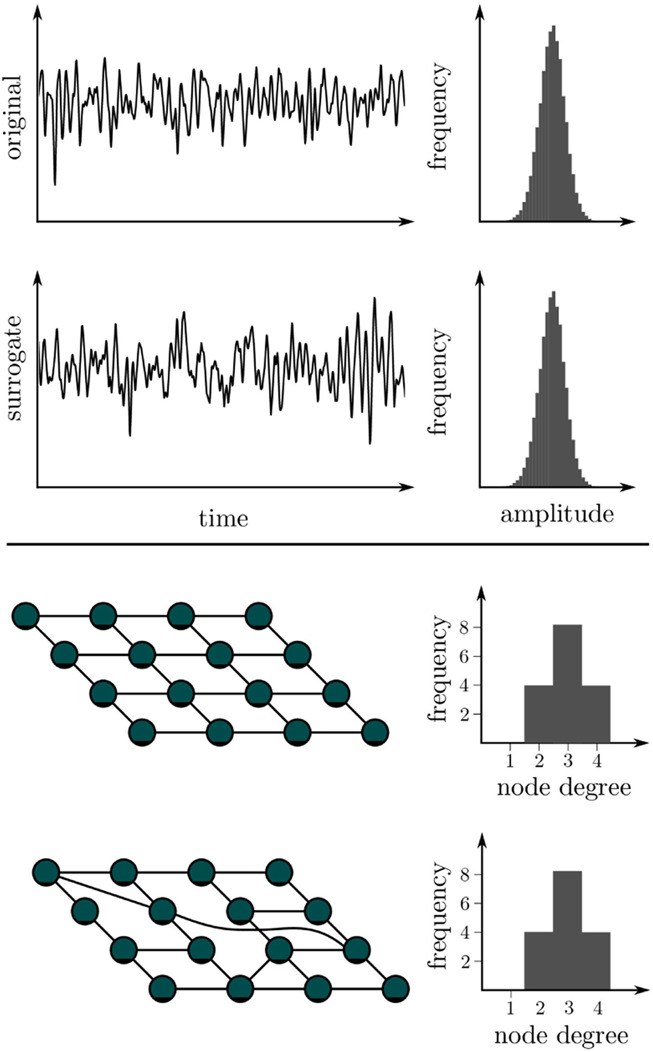

The null hypothesis represents the case for which the obtained findings are consequent to the influencing factors and not to the properties of interest of the investigated system. From this null hypothesis, a pertinent model (the so-called null model) is created, and an ensemble of surrogate data is generated by simulating realizations of the model with Monte Carlo methods (Efron, 1982). In these realizations, all relevant statistical and dynamical aspects of the original data are preserved—except the properties which are tested for. If some discriminating statistics of the original data falls outside the expectation range for the surrogate ensemble, the null hypothesis can be rejected with reasonable confidence (depending on the number of statistically independent constrained realizations). In this case, an alternative hypothesis has to be accepted—i.e., findings are likely due to the properties of the system. However, the surrogate approach does not specify alternative hypotheses nor is it a validation of any specific alternative hypothesis’ accurateness.

For properties of interactions, constrained realizations of the multivariate time series can be generated by randomizing the aspect of a recorded dynamics on which the estimator for the property of an interaction is based (Schreiber, 1998; Schreiber and Schmitz, 2000; Andrzejak et al., 2003a; Paluš, 2007; Rings et al., 2020). However, the associated surrogate techniques are exclusively designed for the strength of an interaction as the formulation of null hypotheses for the direction of an interaction (linkable to properties of time series for an appropriate null model) continues to be an unsolved problem. The same applies to null hypotheses for the functional form of an interaction.

Having estimated the relevant property of interaction for all pairs of brain regions, the values of the estimates then set up an *interaction matrix* $I\in R^{N\times N}$ , where *N* is the number of recorded brain regions.

Subsequently, a binary or weighted and directed or undirected network can be constructed from this matrix.

An *undirected binary network* describes the brain in terms of *connected* or *disconnected* vertices and can be represented by a symmetric adjacency matrix $A\in {\left\{0,1\right\}}^{N\times N}$ (also referred to as functional connectivity). If two vertices *i* and *j* are considered connected, the associated entry $A_{ij}$ is 1 and 0 otherwise. Typically, two vertices are assumed to be connected, if an estimator for the strength of interaction between the associated brain regions exceeds some threshold. There are, however, no commonly accepted criteria for the selection of the threshold [see, e.g., Kramer et al. (2009); Zanin et al. (2012)]. Alternatively, mesoscopic network structures [e.g., minimum spanning tree (Rammal et al., 1986; Stam et al., 2014) or shell or web decompositions (Bröhl and Lehnertz, 2019; Kong et al., 2019)] can be derived from I and used as a binary network.

If it is additionally of interest which brain regions interact how strongly, an *undirected weighted network* provides this information. Represented by a symmetric weight matrix $W\in R^{N\times N}$ (also referred to as functional connectivity), it is possible to again select a threshold to exclude edges with non-significant strengths of interaction. However, in most cases all edges are considered to exist and form a complete network. Typically, elements of the weight matrix are set to be identical to the elements of the interaction matrix, i.e., $W_{ij}=I_{ij}\;\forall \;i,j$ .

A *directed binary network* describes the brain by depicting which brain region drives which other region. Extending the concept of undirected binary networks, directed networks can be described by an asymmetric adjacency matrix $D\in {\left\{0,1\right\}}^{N\times N}$ (also known as effective connectivity), where an entry $D_{ij}$ is 1, if vertex *i* is connected to vertex *j* by a uni-directional edge, and 0 otherwise. If in addition the entry of the inverse direction is also 1 $\left(D_{ij}=D_{ji}=1\right)$ , the vertices are connected by a so-called *bidirectional edge* and are driving each other. A directed edge is assumed to exist, if an indication of direction is strong—e.g., if the value of an estimator for the direction of interaction (or some directionality index derived from the value) exceeds some threshold. Again, the choice of this threshold is arbitrary and there are no commonly accepted criteria for its selection.

Of note, direction of interaction does not generally inform of strength of interaction, and combining both information in a *directed and weighted network* is a not conclusively solved problem. When merging strength and direction of interaction, it is important to remember that both properties are different but not unrelated (Lehnertz and Dickten, 2015). The often-employed interpretation of the modulus of an estimator for the direction of interaction as strength of interaction might not consistently be accurate and can lead to severe misconceptions—particularly for uncoupled or strongly coupled systems (Osterhage et al., 2007b; Paluš and Vejmelka, 2007; Lehnertz and Dickten, 2015; Günther et al., 2022). It is conceptually unclear how weights should be assigned to forward and backward direction of the edges. Strength of interaction is symmetric under exchange of two vertices, while direction of interaction is not. In addition, many concepts employed to estimate properties of interactions can currently not be mapped to each other. The easiest way to avoid resulting problems is to estimate strength and direction of interaction separately but using methods that are based on the same concepts (e.g., phase synchronization or information flow).

There is also no commonly accepted method to utilize functional forms of interaction to derive networks, and doing so would require an abstract, possibly symbolic assignment of edges. Hypothetically, a system’s equations of motion could already be considered a symbolic network were a vertex represents an elementary unit’s self-dynamics function and an edge represents a coupling function. However, how to interpret the multitude of possible functions involved as a network’s component is highly ambiguous.

### 3.3 Properties of a functional brain network

Having derived a functional brain network from observed data, the next task is to characterize the network’s properties and internal organization. While there are a multitude of different network metrics based on concepts and methods from graph theory [see, e.g., Rubinov and Sporns (2010); Newman (2018); Cimini et al. (2019) for an overview; Box 4], they are predominantly defined for undirected (binary or weighted) networks and each metric reflects specific topological or spectral network properties. Network metrics for directed networks are still subject of current research. Two necessary concepts for a characterization of a network are *distance* and *shortest paths*. A *path* is the collection of edges that need to be traversed to reach a constituent starting at another one, and the respective *path length* (which is equivalent to the distance) is either the number of edges that are traversed in case of a binary network, or the sum of the inverse weights of these edges in case of a weighted network. There are multiple paths between every constituents, and shortest paths are the ones whose path lengths are minimal. Generally, network metrics can be categorized according to the network scale for which they are defined—i.e., from the global scale encompassing the whole network to the local scale of single vertices and edges.

BOX 4 **Properties of a network can be assessed with different local-to-global network metrics. Local network metrics (top) assess how individual vertices or edges are integrated into the larger network. These metrics can also be used to determine the importance (centrality) of vertices and edges for the network on the basis of objective criteria. For example, a vertex with a high degree (or strength in case of a weighted network) has a strong influence on the network; conversely, the influence of the network on this vertex can be estimated. Betweenness centrality can be used, e.g., to rate the importance of a vertex (or an edge) for the flow of information in a network. Since a vertex (edge) with a high betweenness centrality is traversed by a large number of paths, it acts like a bottleneck in the network. Global network metrics (bottom) evaluate a network as a whole. For example, if the mean value over all local clustering coefficients of a network takes on a high value, then vertices are closely connected to their neighboring vertices (clique formation) In a network whose average shortest path length is large, vertices are only weakly connected with their neighboring vertices; the network tends to break up into different regions. A (dis-)assortative network, vertices tend to connect with other vertices that are (dis-)similar in some way.**

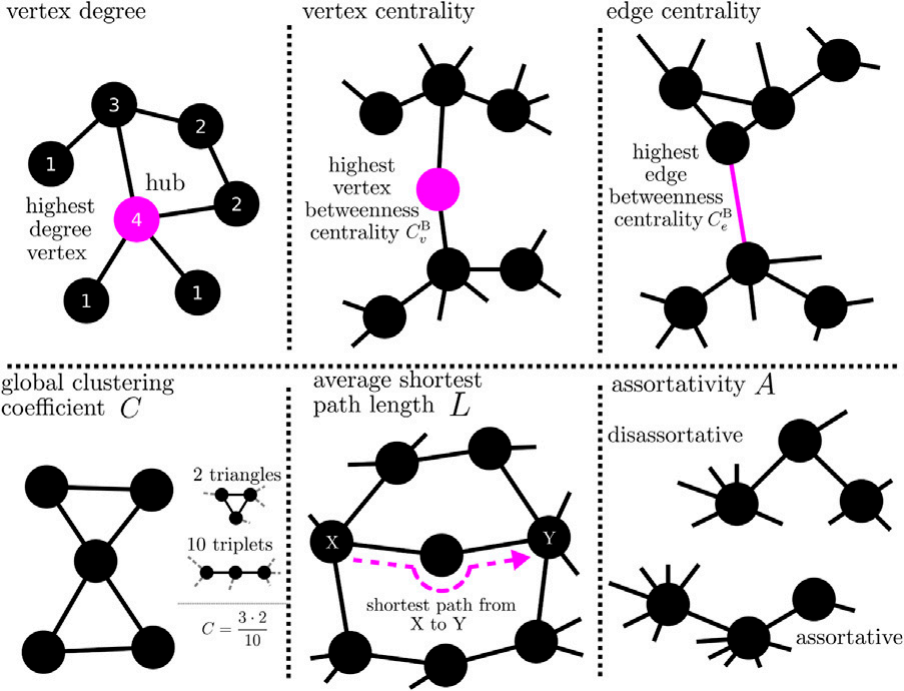

#### 3.3.1 Global scale

On the global scale, network metrics describe the arrangement of vertices and edges according to topological concepts such as *transitivity* [vertices that are connected to two other vertices that are connected themselves; evaluated with, e.g. (global) clustering coefficient (Watts and Strogatz, 1998)], *efficiency* [information or mass transport in a network is facilitated by short paths; e.g., average shortest path length (Newman, 2001)], or *assortativity* [vertices with the same or similar properties are connected preferentially (Newman, 2002)].

In greater detail, the global clustering coefficient assess the number of closed triplets in relation to the total number of triplets in the network and characterizes a network’s functional segregation; segregation decreases with increasing values of the coefficient, however both excessive large or small values indicate a breakdown of segregation. The average shortest path length characterizes a networks functional integration; the shorter the path, the more integrated is the network (cf. Box 5). Assortativity characterizes the mixing of vertices with the similar properties (typically assessed with local network metrics) through being connected (Newman, 2003; Bialonski and Lehnertz, 2013); if edges preferentially connect vertices with a similar (dissimilar) property, such networks are called assortative (disassortative). Disassortative networks are more vulnerable to perturbations and appear to be easier to synchronize than assortative networks (Motter et al., 2005; di Bernardo et al., 2007).

BOX 5 **Following Tononi et al. (1994), integration can be defined as an effective global cooperation between different subsystems (brain regions). Segregation can be defined as the decomposition of a system into sufficiently independent specialized processing subsystems.**

Additionally, methods from linear algebra can be employed to investigate spectral properties of a network’s associated adjacency or weight matrix, which then can be related to a network’s emergent global dynamics [for an overview see (Atay et al., 2006; Comellas and Gago, 2007)]. *Synchronizability*, e.g., characterizes the stability of a global synchronization state, i.e., a network’s propensity to synchronize (Barahona and Pecora, 2002). However, for an interpretation of synchronizability of human brain networks, one should keep in mind that some of this metric’s fundamental assumptions are not fulfilled in this system—for synchronizability, it is assumed that all vertices are associated with dynamics of the same type with largely identical control parameters which strongly disagrees with the spatial heterogeneity of brain dynamics (Papo and Buldú, 2019).

Although it is possible to compensate for this heterogeneity to some degree (while accepting other constraining assumptions) (Sun et al., 2009; Nishikawa and Motter, 2010; Zhang and Motter, 2017), a global synchronization state is fortunately not achieved in the brain (and would indicate complete dysfunction)—an inveterate conceptional issue. Consequently, synchronizability should only ever be treated as an indicator of stability properties of a network’s associated dynamics instead of face value.

Global network metrics can also be used to define indices that are thought to be specific to the network’s topology. For example, global clustering coefficient and average shortest path are often merged to assess whether a given network has a small-world-like topology or not (Bassett and Bullmore, 2006), and this property has been reported for both structural and functional, physiological and pathophysiological brain networks in the past (Reijneveld et al., 2007; Bullmore and Sporns, 2009; Stam, 2014). These findings, however, are strongly contested (Bialonski et al., 2010; Hlinka et al., 2012; Hilgetag and Goulas, 2016; Papo et al., 2016) due to the identification of various factors that can influence the characterization of networks. Confusingly, the literature frequently refers to various metrics and underlying concepts with different, sometimes overlapping names—e.g., the metric “average shortest path length” and the metric “average communication efficiency” are identical and both are indicators of the conceptual “efficiency” of a network.

#### 3.3.2 Local scale

On the local scale, network constituents (vertices as well as edges), can be characterized with so-called centrality metrics. These metrics evaluate a constituent according to the various roles it can play in a network [for an overview see, e.g., Koschützki et al. (2005); Kuhnert et al. (2012); Bröhl and Lehnertz (2019)]. Typically, centrality metrics are based either on the strength with which a constituent is connected to the rest of the network (*strength-based*; e.g., strength centrality or eigenvector centrality) or on their involvement in the organization of shortest paths in a network (*path-based*; e.g., betweenness centrality or closeness centrality). Constituents with high strength-based centrality values are typically considered to affect (and to be affected by) the rest of the network more strongly than constituents with smaller values and are often called *hubs*. Constituents with high path-based centrality values are thought to be important for information or mass transport phenomena on networks, e.g., by being bridges connecting remote network regions (high betweenness centrality) or by reaching other constituents via especially short shortest paths (high closeness centrality).

Local network metrics are also frequently used to assess importance of network constituents by establishing rankings of vertices or of edges (Ghoshal and Barabási, 2011; Lü et al., 2016; Liao et al., 2017). While many studies concentrate on the most important constituents (i.e., the vertex or edge with the highest centrality value), information from the full spectrum of centrality values can be educational when considering the network’s complete internal organization.

#### 3.3.3 Intermediate scale

Extensions of network metrics to an intermediate, mesoscopic scale are subject of current research. In principle, sub-networks can be identified via, e.g., community or module detection (Fortunato and Hric, 2016) or shell or web decompositions (Bröhl and Lehnertz, 2019; Kong et al., 2019) on this scale and then characterized with global or local metrics instead of the whole networks. However, most sub-network identification schemes are themselves based on local network metrics, which might lead to an overemphasis on the concept behind the used local metric. Also, the interpretation of such a characterization of sub-networks is hitherto unclear. Alternatively, the relative amounts of so-called graphlets or motifs (smallest sub-networks interpreted as generic building blocks) can be investigated and related to theoretical arguments about the roles of these objects in a network (Newman, 2006; Alon, 2007; Ribeiro et al., 2021).

In principle, estimating network metrics can be affected by the same adverse influences that also affect the estimation of properties of interactions—either by error propagation or by influencing a researcher’s decision about how to derive the network based on preliminary results (e.g., a higher average strength of interaction might bias the decided-on level of a threshold for a binary network). Especially, oversampling (effectively recording the same dynamics multiple times) and common sources (due to, e.g., referential recording) can lead to misinterpretations (Porz et al., 2014) since especially estimators for strength of interaction identify largely identical time series as an indication of very strong interactions, which in turn influence, e.g., strength-based centrality metrics. In addition, statistical uncertainties from the estimation of properties of interactions can be amplified in unexpected ways when they are merged into network metrics. In some of these cases, so-called *network surrogates* (cf. Box 3) can be employed to improve reliability and reduce adverse influences (Ansmann and Lehnertz, 2011; 2012; Wiedermann et al., 2016; Stahn and Lehnertz, 2017; Váša and Mišić, 2022). These surrogates are constrained realizations of the investigated network by randomizing edges (or their weights) while preserving selected network properties such as network size, density of edges, or distribution of edge weights. Then, to decide to which extent a metric of a given network is determined by these properties, its value can be compared to the values for surrogates of this network. Associated null hypotheses typically state that the internal network organization assessed by the network metric is random under the constraint of the preserved properties.

Finally, to trace time-dependent changes of a network and its internal organization over time, networks are derived for each of the above-mentioned segments of time series of recordings of brain dynamics. This results in a sequence of snapshot networks—the time-evolving functional brain network—and time series of the various network metrics, which can be again investigated with methods from time series analysis (cf. Figure 1).

### 3.4 Characterizing a time-evolving brain network

In the previous subsection, we critically assessed the construction of a (snapshot) functional brain network from windowed data of observed brain dynamics (such as EEG, MEG, or fMRI) using bivariate time-series-analysis techniques and the characterization of this network using graph-theoretical concepts and methods. Performing such analyses for successive windows of observed data results in a temporal sequence of snapshot functional brain networks together with time series of the networks’ metrics, both at a temporal resolution that results from the duration of an analysis window. The sequence and the respective time series form the basis for in-depth studies of a time-evolving brain network which can potentially provide more detailed information about the network’s temporal fluctuations and its complex interplay with ongoing physiologic activities compared to what can be achieved with snippets of recordings of brain dynamics that usually last only a few tens of seconds. Among others, the temporal fluctuations can inform about the significance of averaged quantities such as mean values of some network metrics (Lehnertz et al., 2017; 2021) that are widely used in the network neurosciences.

The identification of spontaneous or induced (patho-)physiologic changes within a sequence of snapshot networks requires estimating some *distance* or (dis-)similarity between two (not necessarily successive) networks (or adjacency matrices). Finding suitable metrics for such a comparison, however, continues to be a difficult task (Bronstein et al., 2006; Andrade et al., 2008; Muskulus et al., 2009; Dimitriadis et al., 2010; Gao et al., 2010; van Wijk et al., 2010; Mémoli, 2011; De Domenico and Biamonte, 2016; Schieber et al., 2017; Fraiman and Fraiman, 2018; Carpi et al., 2019; Martínez and Chavez, 2019; Hartle et al., 2020; Mheich et al., 2020; Lacasa et al., 2021). Difficulties might even aggravate if network size (number of vertices) and edge density depend on time.

**FIGURE 2:**
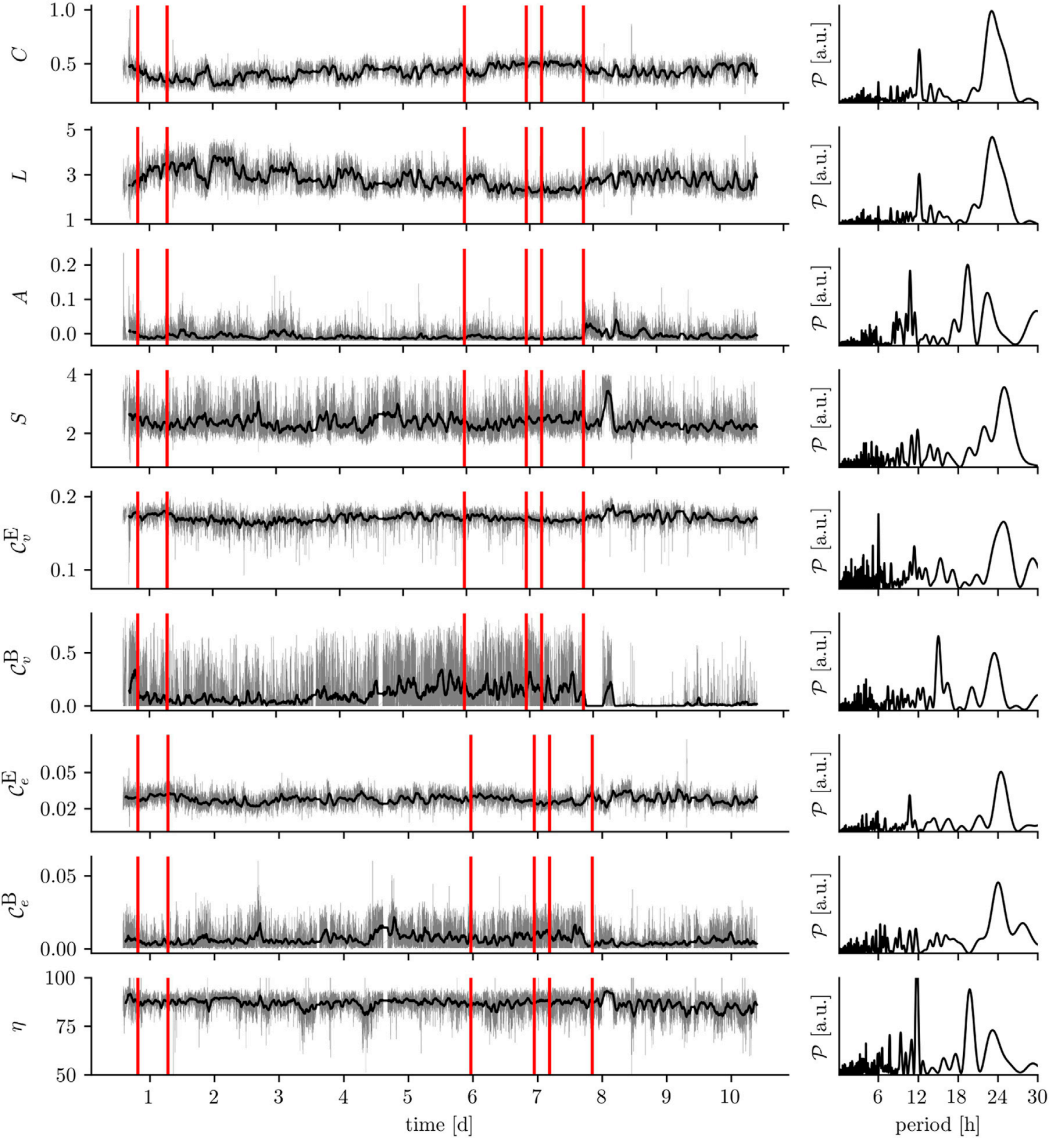
Exemplary time series of various network metrics: global clustering coefficient *C*, average shortest path length *L*, assortativity *A*, synchronizability *S*, eigenvector centrality $C_{v,e}^{\text{E}}$ and betweenness centrality $C_{v,e}^{\text{B}}$ for vertices (v) and edges (e) being most central, on average, and relative number of edges in most important web *η* (Bröhl and Lehnertz, 2019). Time series derived from a multichannel (*N* = 52), long-term (9.8 d) intracranial EEG recording from a subject with a focal epilepsy undergoing presurgical evaluation (the subject signed informed consent that the clinical data might be used and published for research purposes, and the study protocol had previously been approved the by ethics committee of the University of Bonn). Left column shows full (grey) and smoothed time series [black, moving average over 176 windows (1 h) to improve legibility], and tics on x-axis denote midnight. Red lines mark onset of epileptic seizures. Right column shows power spectral density estimates [Lomb–Scargle periodograms (Press and Rybicki, 1989)] of the unsmoothed time series.

An alternative approach is offered by the investigation of time series of the networks’ metrics employing the powerful spectrum of methods from (linear/nonlinear) uni-, bi-, or multivariate time-series-analysis (Bendat and Piersol, 1980; Haykin, 1983; Pikovsky et al., 2001; Kantz and Schreiber, 2003; Reinsel, 2003; Lütkepohl, 2005; Wen and Cheong, 2021; Caligiuri et al., 2023). Statistical (Efron, 1982; Basseville and Nikiforov et al., 1993; Anderson, 2011) as well as Fourier and related analyses (Press and Rybicki, 1989; Huang et al., 1998; Percival and Walden, 2000; Kantelhardt et al., 2001; Bloomfield, 2004) can help to detect anomalies and change points (Aminikhanghahi and Cook, 2017) as well as to assess correlations and periodicities (for an example, see Figure 2). Bi- and multivariate analyses facilitate identification of relationships and interdependencies between time series of different networks’ metrics assessed on the various network scales—from single constituents via communities to the wider network. Before closing this section, we briefly mention another, but so far insufficiently studied analysis approach to investigate time-evolving functional brain networks. It is based on the concept of a multilayer network (Boccaletti et al., 2014; Kivelä et al., 2014; Presigny and Fallani, 2022), which is a complex network structure that consists of multiple networks (e.g., a sequence of snapshot networks). Despite a continuous development of metrics to characterize such a network of networks (Battiston et al., 2014; De Domenico et al., 2015; Nicosia and Latora, 2015; Iacovacci and Bianconi, 2016; Ghariblou et al., 2017; Mandke et al., 2018; Tudisco et al., 2018; Zaoli et al., 2021), applications in the neurosciences and related fields mostly center around frequency-based decompositions or structural and functional decomposition (De Domenico, 2017; Buldú and Porter, 2018; Vaiana and Muldoon, 2020). Due to a number of fundamental problems that arise with this approach, a meaningful interpretation of multilayer brain networks is still to be explored (Buldú and Papo, 2018; Mandke et al., 2018).

## 4 The time-evolving epileptic brain network: What have we learned so far?

### 4.1 The time-evolving epileptic brain network during seizures

Various studies reported increased global clustering coefficients and—although to a lesser extent—average shortest path lengths of time-evolving epileptic brain networks during focal and primary generalized seizures [see, e.g. (Ponten et al., 2007; Kramer et al., 2008; Schindler et al., 2008; Ponten et al., 2009; Kramer et al., 2010)] compared to the seconds before or after a seizure. For 100 focal seizures from 60 people with epilepsy, this observation could be made irrespective of their anatomical onset location (Schindler et al., 2008). If investigated with high temporal resolution (Schindler et al., 2008; Kramer et al., 2010), both network metrics exhibited a concave-like temporal evolution which points to a movement from a more random toward a more regular and more segregated and then back toward a more random functional topology of the epileptic brain network (cf. Box 6). A similar evolution was also observed for time-evolving epileptic brain networks during status epilepticus (Kuhnert et al., 2010).

BOX 6 **Schematic change of exemplary global network metric (here: global clustering coefficient and average shortest path length) and changes of the functional topology during seizure.**

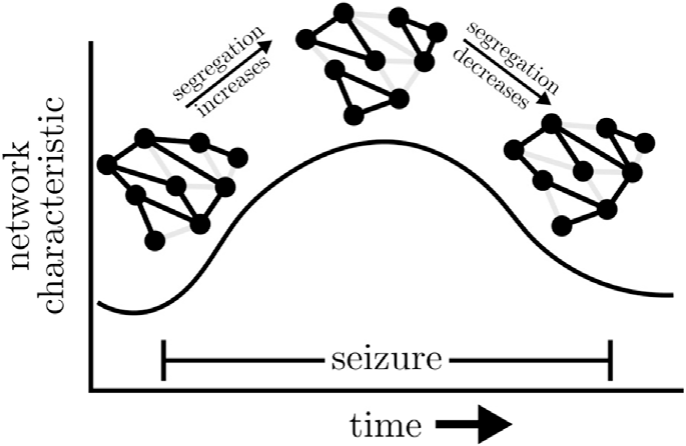

Some authors (Ponten et al., 2007; Kramer et al., 2008; 2010) interpreted the evolution to reflect the small-world topology of short paths and high clustering (Watts and Strogatz, 1998), which is thought to support efficient communication between brain regions at the lowest energetic cost under physiological and pathophysiological conditions (Bassett and Bullmore, 2006; Reijneveld et al., 2007; Bullmore and Sporns, 2012; Stam, 2014). The validity of such an interpretation, however, continues to be matter of considerable debate (Bialonski et al., 2010; Gerhard et al., 2011; Hlinka et al., 2012; Hilgetag and Goulas, 2016; Papo et al., 2016; Hlinka et al., 2017; Zanin et al., 2018) given a large number of factors that have been identified to impact on global clustering coefficient and average shortest path length.

For the same 100 focal seizures from 60 people with epilepsy mentioned above, Bialonski and Lehnertz (2013) reported on a concave-like temporal evolution of assortativity, with a more assortative topology during seizures than during the seconds before or after a seizure. Interestingly, assortativity decreased already prior to seizure end. An increasing assortativity indicates a division of the evolving epileptic brain network into groups of brain regions that are only sparsely interconnected, if at all. Such networks have a comparatively resilient core of mutually interconnected high-degree vertices as has been observed by Zubler et al. (2015) for 198 seizures from 27 people with epilepsy. This core makes epileptic brain networks during seizures quite robust against perturbations, which might explain the mixed success of active brain stimulation to interrupt seizures (Morrell, 2006; Hirsch and Schulze-Bonhage, 2023).

It is important to note that the aforementioned topological network alterations are not accompanied by an increased network synchronization [see, e.g., Schindler et al. (2007b; a); Cash (2013); Majumdar et al. (2014)], which puts into perspective the textbook description of seizures as hypersynchronous events (see also Jiruska et al., 2013). Rather, studies on network synchronization and on the networks’ synchronizability indicate that the changing network topology during seizures is accompanied by an initially decreased network synchronization and decreased stability of the globally synchronized state, both of which increase already prior to seizure end (Schindler et al., 2008; Kramer et al., 2010; Khambhati et al., 2015; Frassineti et al., 2021; Rungratsameetaweemana et al., 2022; Salami et al., 2022). These synchronization phenomena may thus be considered as an emergent (network-topology-mediated) self-regulatory mechanism for seizure termination (Lado and Moshé, 2008; Kramer et al., 2012). It is also important to note that the seizure-related topological network alterations are similar across different types of epilepsies, seizures, medication, age, gender, and other clinical features (see also Haneef and Chiang, 2014). This might point to a common mechanism underlying seizure dynamics in the epileptic brain network (Lehnertz et al., 2014).

In addition to these global aspects of time-evolving epileptic brain networks, several studies investigated the role that network vertices and edges play in seizure evolution (Kramer et al., 2008; Wilke et al., 2011; Varotto et al., 2012; Burns et al., 2014; Geier et al., 2015a; Zubler et al., 2015; Goodfellow et al., 2016; Geier and Lehnertz, 2017b; Bröhl and Lehnertz, 2019; Bröhl and Lehnertz, 2022). Employing various centrality and other metrics to characterize a constituent’s importance for seizure dynamics, most studies reported these metrics to exhibit a high temporal variability as seizures evolve, both inter- and intraindividually. While some studies reported most important vertices (if based on a metric’s temporal mean) to coincide with the clinically defined seizure onset zone (SOZ), other studies could not confirm such a relationship. Rather, network vertices associated with brain regions deemed unaffected by the pathology and more recently also edges (Bröhl and Lehnertz, 2019; Bröhl and Lehnertz, 2022) that functionally connect these vertices were reported as most important during the course of a seizure. If at all, vertices that can be associated with the SOZ gained importance towards the end of a seizure (Burns et al., 2014; Geier et al., 2015a; Zubler et al., 2015).

It remains to be investigated which factors could have led to these inconsistencies, apart from methodological issues (Geier and Lehnertz, 2017b). Nevertheless, the observation of network constituents that are most important during seizures but appear to be unrelated to pathological brain tissue not only underlines the significance of the concept of an epileptic network but also puts into perspective the role of the epileptic focus in seizure dynamics (see also Paz and Huguenard, 2015). One might speculate whether such network constituents represent potential targets for focused therapeutic interventions.

### 4.2 The time-evolving epileptic brain network during the pre-seizure state

Despite the well-known observation that *vulnerability to seizure activity in any one part of the network is influenced by activity everywhere else in the network, and that the network as a whole is responsible for the clinical and electrographic phenomena that we associate with human seizures* (Spencer, 2002), we still lack a sufficient quantitative assessment of the time-evolving epileptic brain network’s metrics (from the local to the global network scale) that would help to improve understanding of how the network generates seizures (Kuhlmann et al., 2018; Lehnertz, 2021; Lehnertz et al., 2023) as well as other pathophysiological phenomena (Weiss et al., 2022).

Nevertheless, first indications for certain network reconfigurations to promote the formation of a pre-seizure state could be derived from retrospective studies that investigated macroscopic metrics of time-evolving epileptic brain networks. Kuhnert et al. (2010) analyzed more than 2,100 h of continuous intracranial EEG recordings from 13 subjects with epilepsy during which 75 focal onset seizures and one status epilepticus occurred. From the time series of global clustering coefficients and average shortest path lengths, the authors observed the distributions of these metrics from pre-ictal periods [assumed duration: 4  h; cf. Mormann et al. (2006)] to significantly deviate from the respective distributions of metrics derived from inter-ictal data. Both these global metrics of network structure attained higher values (on average) pre-ictally in the majority of subjects, which the authors interpreted as indications for a loss of functional long-range connections during the pre-ictal period. Geier et al. (2015b) performed similar analysis for assortativity (here: degree-degree correlations) based on more than 1,000 h of continuous intracranial EEG recordings from seven subjects with epilepsy during which 16 focal onset seizures occurred. Pre-ictally, a slightly less assortative mixing of time-evolving epileptic brain networks was observed, which might indicate these networks to be less robust against (endogenous and/or exogenous) perturbations. Both, Kuhnert et al. (2010) and Geier et al. (2015b) stressed, however, the strong influence of daily rhythms seen in the time series of the investigated network metrics (cf. Section 4.3) that would need to be taken into account to avoid misinterpretations (see also Takahashi et al., 2012).

More recently, Rings et al. (2019a) used the network approach to develop a time-series-analysis technique that allows tracing resilience of a networked dynamical system (Fischer et al., 2022), such as the brain. The authors investigated more than 3,200 h of continuous intracranial EEG recordings from 43 subjects with epilepsy during which 112 focal onset seizures occurred. They observed the distribution of the network resilience estimator (dynamical resistance) from 4 h pre-ictal periods to significantly deviate from the respective distribution of the metric derived from inter-ictal data. The achieved high, above-chance-level predictive performance [evaluated with seizure time surrogates (Andrzejak et al., 2003b)] of dynamical resistance would qualify this resilience estimator for seizure-prediction studies. In passing, we note that other estimators of resilience such as those related to the concept of critical slowing down failed to achieve a sufficient predictive performance (Milanowski and Suffczynski, 2016; Wilkat et al., 2019; Hagemann et al., 2021) rating this concept overly simplistic for the human epileptic brain. Interestingly, dynamical resistance increased in the hours prior to the vast majority of seizures. Although one would expect intuitively resilience to decrease in order to facilitate the generation of a seizure, the authors speculated that the reduced effectiveness of antiseizure medication may account for the observed increase. One might also speculate [see the discussions in Frei et al. (2010); Zaveri et al. (2020)] that a pre-ictally increased brain resilience could also reflect the epileptic brain network’s ability to efficiently defy control because of its intrinsic plasticity and adaptiveness. In this context, epilepsy may be viewed as a “learned” disease and seizures as an abnormal learned response to recurrent perturbations—such as seizures (Turrigiano, 1999; Hsu et al., 2008; Lignani et al., 2020; Issa et al., 2023).

In addition to studies on pre-seizure-state-related alterations of functional segregation and integration as well as of robustness of the time-evolving epileptic brain network, further in-depth insights into pre-seizure network reconfigurations could be achieved with investigations of time-dependent changes of properties of the network’s vertices and edges. Tauste Campo et al. (2018) investigated non-continuous intracranial EEG recordings from 10 subjects with epilepsy and used an averaged vertex eigenvector centrality to characterize network state variability. The authors observed network states to become less variable a few hours preceding a global functional connectivity reduction before seizure onset [cf. Mormann et al. (2000; 2003); Kuhlmann et al. (2010); Lehnertz et al. (2016)].

Analyzing retrospectively more than 3,200 h of continuous intracranial EEG recordings from 38 subjects with epilepsy during which 97 focal onset seizures occurred, Rings et al. (2019b) observed distributions of vertex centrality (strength and betweenness centrality) and edge weight from 4 h pre-ictal periods to significantly deviate from the respective distributions of these local network metrics derived from inter-ictal data. The authors reported high, above chance level predictive performance for these deviations and observed that most brain regions (vertices) whose dynamics carried predictive information were connected by most of the edges whose time-dependent weight changes carried predictive information. These vertices, however, never played a central role in the investigated time-evolving epileptic brain networks. More importantly, these vertices were entirely associated with brain regions far off the clinically defined SOZ.

Based on these observations, the authors proposed a scenario for the generation of seizure precursors in a time-evolving epileptic brain network (cf. Box 7): endogenous and/or exogenous factors trigger a rearrangement of the network’s path structure which eventually leads to a formation of bottlenecks in brain regions deemed unaffected by the pathological process which in turn impairs physiologic brain communication [cf. (Avena-Koenigsberger et al., 2018)]. These brain regions, being part of the large-scale epileptic brain network, generate and sustain normal, physiologic brain dynamics during the inter-ictal intervals. Moreover, they also appear to efficiently control the dynamics of vertices related to the SOZ (Lehnertz and Dickten, 2015; Dickten et al., 2016; Johnson et al., 2023).

BOX 7 **The path-structure (i.e., layout of the paths in the network) of the functional network is altered prior to seizure, culminating in the forming of bottlenecks. Black networks represent the average functional network in the respective interval. Dotted edges indicate lower interaction strength.**

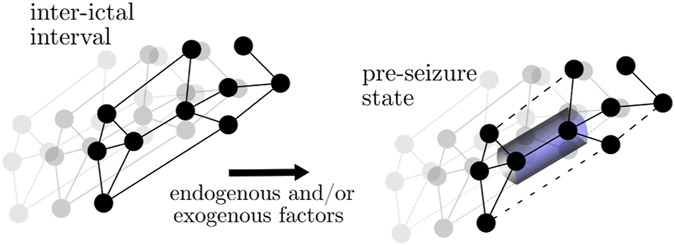

Fruengel et al. (2020) investigated retrospectively continuous intracranial EEG recordings that were part of previous studies (Lehnertz et al., 2016; Rings et al., 2019b) and employed different strength- and path-based vertex centrality metrics (strength, eigenvector, closeness and betweenness centrality) to further improve understanding of local and global reconfigurations of the time-evolving epileptic brain network during the pre-seizure period. The authors observed distributions of vertex centrality from 4 h pre-ictal periods preceding 99 seizures to significantly deviate from the respective distributions of these local network metrics derived from inter-ictal data. As a result of high, above chance level predictive performances for these deviations in various brain regions, they proposed several major scenarios for a pre-seizure reconfiguration of the time-evolving epileptic brain network. With these scenarios, pre-seizure changes in the network are not necessarily confined to specific brain regions. Rather, local and global reconfigurations of the time-evolving epileptic brain network appear to affect virtually all network constituents, i.e., various vertices (brain regions) and the edges (functional connections) between them. Focusing on the pre-seizure changes in degree and betweenness centrality of vertices associated with the SOZ, Sumsky and Greenfield Jr (2022) recently reported similar findings in the seconds prior to 143 seizures from 20 subjects with epilepsy and investigating a comparable amount of continuous intracranial EEG recordings (more than 3,300 h).

### 4.3 The time-evolving epileptic brain network during seizure-free intervals

If a time-evolving epileptic (and non-epileptic) brain network is derived from continuous multiday, intracranial or scalp-recorded EEG, virtually all time series of the network’s metrics—from local to global—exhibit large fluctuations over time which appear to be partly periodic (cf. Figure 2). These periodicities can be correlated with the circadian and various ultradian rhythms (Kuhnert et al., 2010; Takahashi et al., 2012; Lehnertz et al., 2014; Geier et al., 2015b; Geier and Lehnertz, 2017a; Lehnertz et al., 2017; Rings et al., 2019a; Chiosa et al., 2019; Mitsis et al., 2020; Healy et al., 2021; Lehnertz et al., 2021; Bröhl et al., 2023), which are also thought to modulate seizure risk (Bernard, 2021; Karoly et al., 2021) and seizure dynamics (Schroeder et al., 2020). Despite these large periodic fluctuations, additional alterations of network metrics can be observed on shorter time scales (some tens of seconds to few minutes) typically covered in clinical and research studies. These (mostly time-averaged) alterations of metrics seen in epileptic brains clearly differ from those seen in healthy ones (Chavez et al., 2010; Horstmann et al., 2010; Vlooswijk et al., 2011; Zhang et al., 2011; Richardson, 2012; Caciagli et al., 2014; Haneef and Chiang, 2014; Stam, 2014; Chiang et al., 2015; Foit et al., 2020; Pegg et al., 2020; Slinger et al., 2022). Importantly, they also differ between different types of epilepsy (Lee et al., 2006; Barzegaran et al., 2012; Bartolomei et al., 2013; Chowdhury et al., 2014; van Diessen et al., 2016; Rosch et al., 2018; Kinney-Lang et al., 2019; Lopes et al., 2019; Marino et al., 2019; Ahmadi et al., 2020; Pegg et al., 2020; Woldman et al., 2020; Pegg et al., 2021; Slinger et al., 2022; Tufa et al., 2022). Identifying alterations of network metrics is thus thought to contribute to improving differential diagnosis, treatment, surgical planning, and estimation of prognosis.

This perspective is further supported by research findings of alterations of network metrics due to factors that are known to modulate the epileptic process such as cognition (Kuhnert et al., 2013; Shine, 2019), treatment with antiseizure medication (Anderson et al., 2020; Hatlestad-Hall et al., 2021) and with neuromodulation such as deep brain stimulation (Khaledi-Nasab et al., 2022; Vetkas et al., 2022), responsive neurostimulation (Piper et al., 2022), and vagus nerve stimulation (VNS) (Fraschini et al., 2014). As regards the latter, investigations in larger groups of subjects with epilepsy and in healthy controls (Rings et al., 2021; von Wrede et al., 2021; 2022a; b) demonstrated that short-term, non-invasive transcutaneous auricular VNS can induce small but measurable immediate and enduring alterations of global metrics of the time-evolving epileptic brain network while leaving its local metrics essentially unchanged. The differential alterations of local and global network metrics can be understood using the model of stimulation-mediated stretching and compression of the time-evolving epileptic brain network proposed by Rings et al. (2021). This model takes into account the changes of the network’s path structure (average shortest path length) and of its tendency to form tightly knit groups of vertices (global clustering coefficient) as well as the centrality (importance) hierarchies of vertices and edges to characterize stimulation-mediated modifications of the larger network. The authors conjectured that these topology-modifying stretching and compression effects likewise impact on the network’s assortativity and synchronizability, thereby enhancing its robustness and stability.

Recently, Lehnertz et al. (2023a) reported on a similar reconfiguration and modification of networks together with their stability and robustness properties in a group of 20 subjects with and without epilepsy upon a short-term manual visceral-osteopathic stimulation of the vagus nerve at the abdomen. This finding may add to the current discussion on the importance of the gut-brain axis in the treatment of epilepsy (Ding et al., 2021; Sinha et al., 2022) and to further enhance our understanding of how multiple organs in the human body dynamically interact as a network and integrate their functions to generate (patho-)physiological states (Ivanov, 2021).

### 4.4 A model for the temporal evolution of the epileptic brain network

**FIGURE 3:**
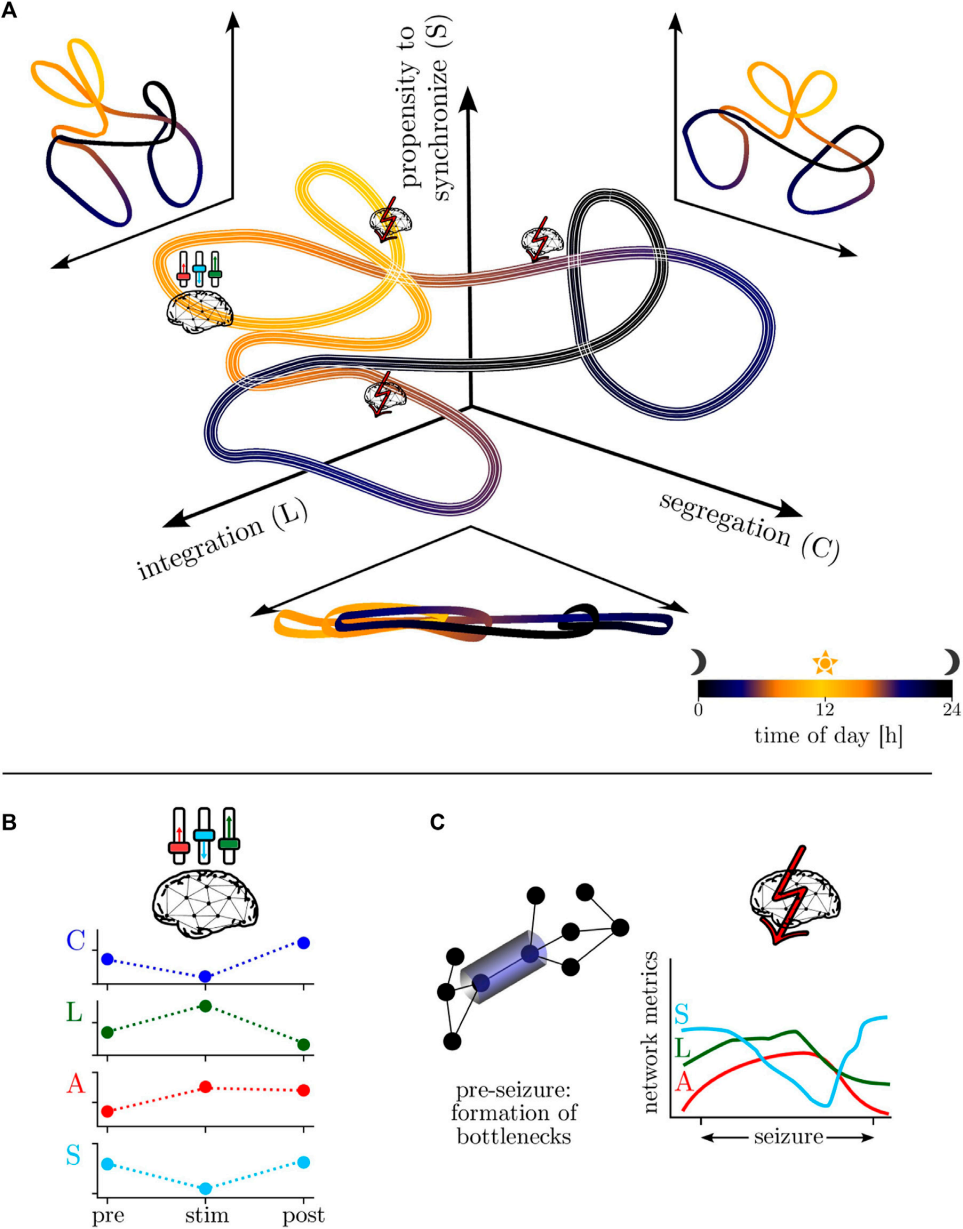
**(A)** Model of the temporal evolution of the epileptic brain network in an abstract “phase-space”—spanned by the networks’ properties of segregation, integration, and propensity to synchronize—over the day-night cycle (color-coded). Line thickness encodes infradian variability. **(B)** Schematic of neurostimulation-induced modifications of global network properties (global clustering coefficient *C*, average shortest path length *L*, assortativity *A*, synchronizability *S*; local ones remain essentially unchanged). **(C)** Schematic of local network modifications (here: formation of bottlenecks) related to pre-seizure dynamics and of global modifications related to seizure dynamics.

Summarizing the findings achieved so far, we propose a model for the temporal evolution of the epileptic brain network (cf. Figure 3A). To this end, we consider an abstract “phase-space” that is spanned by the networks’ global clustering coefficients *C*, average shortest path lengths *L*, and synchronizabilities *S* to capture the diurnal variation of segregation, integration, and the networks’ propensity to synchronize (although they are correlated, we use *C* and *L* to facilitate readability). The networks’ motion in this space is largely dominated by the circadian rhythm (with a period length of about 24 h; cf. Figure 2), with a comparably lower (higher) segregation (integration) as well as an increased propensity to synchronize during daytimes. This global motion is modulated by ultradian rhythms with period lengths around 12 h and shorter, seen during both night- and daytimes (cf. Figure 2). These modulations likely reflect different sleep/vigilance states and their accompanying modifications of critical network properties [such as segregation and integration (Deco et al., 2015) as well as the propensity (or vulnerability) to be synchronized by an admissible input activation] may account for the well-known fluctuations of epileptic activities and seizure occurrence (Spencer et al., 2016; Khan et al., 2018). Interestingly, the networks’ motion is, in general, only sparsely modulated by comparably short-lasting exogenous (e.g., neurostimulation; cf. Section 4.3 and Figure 3B) and endogenous perturbations (seizures; cf. Section 4.1 and Figure 3C). Nevertheless, the specific manner of these modulations provides novel insights into the effectiveness of neurostimulation/-modulation approaches as well as into network mechanisms of pre-seizure dynamics (cf. Section 4.2), seizure generation, spread, and termination.

## 5 Current limitations and potential prospects

Initiated by Susan Spencer’s seminal work on neural networks in human epilepsy, the last two decades have seen quite a number of accomplishments in defining and characterizing the epileptic brain network, including an important change in perspective from a static to a time-evolving network (cf. Li et al., 2017), which better takes into account the fact that epilepsy is a dynamic disease. Nevertheless, much remains to be completed in the coming years, and several issues need to be addressed to advance the field.

### 5.1 Methodological issues

Beginning with collecting the brain’s structure and dynamics, there is a strong need for a recording technique that allows one to capture the multiple spatial and temporal scales of the epileptic process. If different recording techniques are used, there is still the problem of an unambiguous fusion of the different scales (Schevon et al., 2019; Driscoll et al., 2021; Presigny and Fallani, 2022). Likewise, suitable strategies to avoid spatial and temporal oversampling of brain dynamics are urgently needed since oversampling can lead to severe misinterpretations of network characteristics (Bialonski et al., 2010; Zalesky et al., 2010; Bialonski et al., 2011; Joudaki et al., 2012; Montes-Restrepo et al., 2014; Porz et al., 2014; Puce and Hämäläinen, 2017; Conrad et al., 2020; Vorderwülbecke et al., 2020; Iivanainen et al., 2021; Allouch et al., 2023). It remains to be shown whether recent developments of edge/vertex removal strategies (Bellingeri et al., 2020; Bröhl and Lehnertz, 2023) [or, in case of undersampling, techniques to predict edges (Zhou, 2021) and to detect hidden vertices (Su et al., 2012)] can help to avoid misinterpreting characteristics of the time-evolving epileptic brain network.

Open questions related to deriving functional brain networks from recordings of brain dynamics were recently summarized by Korhonen et al. (2021). Beyond that, we note that defining network edges from properties of an interaction between pairs of brain regions derived from their dynamics is predominantly based on bivariate time-series-analysis techniques that assume a deterministic mechanism behind interactions and any stochastic aspect is treated as mere measurement noise. Recent developments in characterizing two-dimensional stochastic processes (Rydin Gorjão et al., 2019; Aslim et al., 2021) based on the Kramers–Moyal expansion might provide novel insights into stochastic interactions in the future. Likewise, a further improved characterization of the temporal structure of the brain’s dynamics could be achieved with bivariate ordinal time-series-analysis techniques (Lehnertz, 2023) that allow one to assess both strength and direction of an interaction. Moreover, while there is an increasing interest in so-called higher-order interactions (Battiston et al., 2021; Bianconi, 2021; Boccaletti et al., 2023) (interactions between more than pairs of brain regions), it remains unclear how to estimate the relevant higher-order interactions from time-series data and what advantages such *hyper networks* will provide aside from theoretical arguments.

In Sections 3.3 and 3.4, we presented network metrics and analysis tools to characterize a time-evolving brain network and briefly discussed their pros and cons. Most concepts and metrics were initially developed to characterize static networks and are not immediately transferable to a time-evolving network. Recently proposed analysis tools for time-dependent networks [see, e.g., De Domenico (2017); Thompson et al. (2017); Salcedo-Sanz et al. (2022)] are either not straightforward to convert to weighted and complete networks (as in case of EEG-derived functional brain networks) or wait for proof of their suitability for characterizing a time-evolving epileptic brain network. In general, concepts and metrics proposed and used to characterize static networks need to be redefined or appropriately extended to time-evolving networks, also to account for the impact of time ordering on causal relationships in weighted and directed networks.

Additional in-depth insights into the complex behavior of a time-evolving epileptic brain network prior to and during seizures could be achieved with novel concepts and tools to characterize signal propagation in complex networks (Hens et al., 2019; Ji et al., 2023) and synchronized dynamics of time-varying networks (Ghosh et al., 2022). Considering the recent promising developments of centrality concepts and metrics to characterize properties of edges as well as the edges’ time-varying role in the larger network (Bröhl and Lehnertz, 2019; Bröhl and Lehnertz, 2022; Contisciani et al., 2022; Altafini et al., 2023), adopting an edge-centric perspective (cf. Faskowitz et al., 2022; Novelli and Razi, 2022) could lead to a further improved understanding of the time-evolving epileptic brain network and its control (Sinha et al., 2022; Lehnertz et al., 2023; Frauscher et al., 2023).

### 5.2 Conceptual issues

A variety of computational/mathematical models for epilepsy have been proposed [see, e.g., Lytton (2008); Holt and Netoff (2013); Depannemaecker et al. (2021); Pathak et al. (2022)], mostly based on concepts from dynamical systems theory. The majority of these models focuses on seizure dynamics (initiation, spread, termination) only, and the network character of the disease is only rarely taken into account (Kalitzin et al., 2019; Goodfellow et al., 2022). Seizure-like events (states of synchronous rhythmic activity), however, may also emerge spontaneously—i.e., without a change in control parameters—from an oscillator network with some balance between regular and random topology (Rothkegel and Lehnertz, 2014; Ansmann et al., 2016; Gerster et al., 2020; Anesiadis and Provata, 2022; Wu et al., 2022). Other mechanisms behind tipping phenomena include noise-, rate-, and shock-induced tipping [see, e.g., Ashwin et al. (2012); Feudel et al. (2018); Ritchie et al. (2023); Swierczek-Jereczek et al. (2023)]. A better understanding of how seizures emerge from an aberrant, time-evolving epileptic brain network would profit from considering, e.g., critical transition scenarios other than bifurcation-induced tipping which may be too simplistic for the human epileptic brain (Wilkat et al., 2019). Neither of these phenomena require any change of the system’s stability, and various time-series-analysis techniques have been proposed to identify early warning indicators (George et al., 2021; Heßler and Kamps, 2022).

Similarly, a better understanding of the longer time scales of brain dynamics that govern the recurrence of seizures would profit from considering mechanisms that can give rise to various long term, fluctuating behavior. We here mention switching phenomena related to the different types of intermittency (Perez Velazquez et al., 1999; Rizzi et al., 2016; Pisarchik et al., 2018), switching in fast-slow systems (Kuehn, 2011) and in heteroclinic networks (Kirst and Timme, 2008; Aguiar et al., 2011; Bick and Field, 2017; Morrison and Young, 2022; Meyer-Ortmanns, 2023) multistability (Lopes da Silva et al., 2003; Takeshita et al., 2007; Rothkegel and Lehnertz, 2009; Breakspear, 2017; Pisarchik and Hramov, 2022), and metastability (Kelso, 2012; Tognoli and Kelso, 2014; Rossi et al., 2023). The validity of such models could be tested if continuous long-term recordings of brain dynamics—covering weeks to months [see, e.g., Weisdorf et al. (2019); Duun-Henriksen et al. (2020)]—would be publically available.

### 5.3 Translational issues

In order to consolidate the network approach into clinical practice, the following issues would need to be tackled.

*Diagnosing epilepsy:* The understanding and treatment of epilepsy requires a clear-cut diagnosis of the possibly underlying disease, allocation of syndromes, and distinction from other neurological and non-neurological diseases, in comparison to a healthy brain. Nevertheless, the techniques currently used routinely only allow a clear-cut diagnosis in, on average, 50% of the subjects (Oto, 2017; Elger and Hoppe, 2018; Amin and Benbadis, 2019), which can probably be related to a number of confounding factors. It can be conjectured that the incorporation of the concept of a time-evolving epileptic brain network into aforementioned differentiation steps can lead to an improved diagnosing and classification of epilepsy, even on a personalized level (Nabbout and Kuchenbuch, 2020).

*Choosing treatment:* Currently, clinical decisions regarding treatment options are primarily guided by the epilepsy syndrome and its burden. Despite several options [e.g., pharmacological treatment (Kwan and Brodie, 2000; Wandschneider and Koepp, 2016; Höller et al., 2018; Höller and Nardone, 2021), surgical treatment (Téllez-Zenteno et al., 2007), nutritional treatment (Pizzo et al., 2022), neurostimulation (Schulze-Bonhage, 2019; Piper et al., 2022); see Lehnertz et al. (2023) for an overview of network-based treatment concepts], the treatment is successful in only about half of the cases. Moreover, from a clinical point of view, the goal of epilepsy treatment is seizure freedom and if this is not possible reduction of seizure frequency and burden of the disease. However, the epileptic brain is not a temporarily disturbed normal brain, and a seizure is not a clinical sign of a transient dysfunction of a normal brain. Therefore, on a conceptual level, the treatment of epilepsy should be addressed more as the treatment of an evolving epileptic brain rather than treating seizures. Further investigation into the network-modulating effects of different interventions—adopted to the time-evolving epileptic brain network—is vital to provide physicians with information about the best and most promising treatment options in individual treatment situations.

*Optimizing and monitoring therapy:* Treating subjects with epilepsy means to achieve a situation in which the subject not only has no seizures, despite the disease epilepsy, but also is able to live an unimpaired life. Impairments result not only from seizures, but also possibly from an underlying structural correlate, and the epilepsy treatment. A thorough clinical action includes therapy monitoring, adapting therapies to the current situation, and preferably acting with foresight in order to avoid anticipatory therapy consequences. However, available data is limited by the subjective perception and possible inadequate sampling of other influencing dynamics and their interactions [e.g., pharmacokinetics, biological rhythms, fluctuations of the endocrinal system (Lehnertz et al., 2020; Healy et al., 2021)], and hence is not yet sufficient to reliably inform such clinical action. Individual tracking the epileptic brain’s network changes over the time—not only during seizures, but also in response to chronic treatment, during everyday activities and therapeutic *ad hoc* interventions—are needed, to unveil the potential for tailored epilepsy treatment. This treatment should be targeted at the time-evolving epileptic brain network and keep it in states in which the subject can live everyday life without impairment. For a treatment to be successful in the long term, it is essential to keep in mind the brain’s adaptivity and learning capabilities, and to modulate them in an appropriate way to achieve a healthier brain network which “unlearned” epilepsy.

## 6 Conclusion

Recognizing epilepsy as a network disease has sparked extensive and expanding research, which led to much progress towards the understanding of the human epileptic brain as well as prediction and control of its dynamics. This has reshaped the comprehension and perception of epilepsy, entailing a paradigm shift from a clinically defined epileptic focus via a spatially and functionally extended epileptic network to a large-scale, time-evolving epileptic brain network, whose changes comprise various temporal and spatial scales. Although such an approach poses a difficult task, the last two decades have been coined by novel insights and progression, ranging from recording the brain’s structure and dynamics at various spatial and temporal scales to constructing functional brain networks and investigating their properties with various innovative and adapted customized tools of analysis. Insights achieved so far regarding the temporal evolution of the epileptic brain network show great potential for clinical translation, progressing and maturing the state-of-art of diagnosis and treatment of epilepsy. Further studies on the temporal-evolution of epileptic and other diseased brain networks in comparison to non-affected brains will help to achieve these goals.
